# Pharmacokinetics, pharmacodynamics, and safety of clevidipine after prolonged continuous infusion in subjects with mild to moderate essential hypertension

**DOI:** 10.1007/s00228-012-1260-3

**Published:** 2012-03-29

**Authors:** William B. Smith, Thomas C. Marbury, Steven F. Komjathy, Mark S. Sumeray, Gregory C. Williams, Ming-yi Hu, Diane R. Mould

**Affiliations:** 1 Volunteer Research Group, University of Tennessee Medical Center, Knoxville, TN USA; 2 Orlando Clinical Research Center, Orlando, FL USA; 3 Eli Lilly and Company, Indianapolis, IN USA; 4 Aegerion Pharmaceuticals, Cambridge, MA USA; 5 The Medicines Company, Parsippany, NJ USA; 6 Projections Research, Phoenixville, PA USA

**Keywords:** Clevidipine, Hypertension, Blood pressure, Antihypertensive, Intravenous, Calcium channel blocker, 1,4-dihydropyridine, Pharmacokinetics, Pharmacodynamics

## Abstract

**Purpose:**

Clevidipine is a rapidly-acting intravenous dihydropyridine antihypertensive acting via calcium channel blockade. This was a randomized, single-blind, parallel-design study of a 72-h continuous clevidipine infusion.

**Method:**

Doses of 2, 4, 8, or 16.0 mg/h or placebo were evaluated in 61 subjects with mild to moderate essential hypertension. IV clevidipine or placebo was initiated at 2.0 mg/h and force-titrated in doubling increments every 3 min to target dose, then maintained for 72 h. Blood pressure and heart rate were measured during infusion, and for 4, 6 and 8 h after termination of infusion, although oral therapy could be restarted at 4 h. Clevidipine blood levels were obtained during infusion and for 1 hour after termination.

**Results:**

Rapid onset of drug effect occurred at all clevidipine dose levels, with consistent pharmacokinetics and rapid offset after 72-h infusion. No evidence of tolerance to the clevidipine drug effect was observed at any dose level over the 72-h infusion. No evidence of rebound hypertension was found for either 4 or 6 h after termination of the clevidipine infusion. At 8 h following cessation of clevidipine, blood pressure was not significantly higher than at baseline. Placebo-treated subjects had blood pressures lower than baseline at 8 h following infusion termination; hence, placebo-adjusted blood pressures tended to be slightly higher than baseline.

**Conclusion:**

This study supports the use of up to 72 h of IV clevidipine therapy for the management of blood pressure, with consistent pharmacokinetic/pharmacodynamic characteristics and context insensitive half-life across the dose ranges evaluated.

## Introduction

Subjects with acute, severe hypertension require intravenous (IV) therapy when rapid, controlled blood pressure (BP) reduction is necessary and the administration of oral agents is not feasible [1, 2]. The ideal therapeutic profile for an IV antihypertensive agent would include rapid action, predictability, easy titratability, short duration after discontinuation, and minimal risk of unwanted drug effects [2–5].

Clevidipine, the latest-generation dihydropyridine calcium channel blocker, lowers BP through arterial-specific peripheral vasodilation without adverse myocardial effects. Owing to its direct effect activity and ultra-short half-life, clevidipine has a rapid onset and offset of effect allowing precise titration to target BP levels and maintenance of tight control over time [6–8]. Following administration, clevidipine exhibits triphasic elimination with the initial phase accounting for the majority (approximately 85–90 %) of the area under the concentration–time curve [8–10]. Metabolism is achieved by the action of esterases in the blood and extravascular tissues, resulting in an initial half-life of approximately 1 min [8–10].

There is limited clinical experience with prolonged (>24 h) continuous infusion of clevidipine. This study evaluated the pharmacokinetics (PK), pharmacodynamics (PD), and safety of continuous clevidipine infusion, including the potential for tolerance during treatment and for rebound hypertension upon discontinuation, over 72 h in subjects with mild to moderate essential hypertension.

## Materials and methods

### Study design

The study was conducted using a randomized, single-blind, placebo-controlled, parallel-group design. There were three study phases: screening/run-in for up to 14 days (Study Days −14 to −1), treatment for 72 h (Days 1–3) and follow-up for 4 days (Days 4–7), for a maximum of 21 days. All eligible subjects were confined to the clinic at least 12 h before treatment. Subjects were randomized to one of four dosing cohorts for continuous treatment over 72 h with clevidipine or placebo. The study was approved by the Institutional Review Board at each study site, and was conducted in compliance with the International Conference on Harmonization (ICH) Good Clinical Practice (GCP) guidelines, and with the Declaration of Helsinki. Written informed consent was obtained from all subjects before the initiation of any study-related procedures.

### Subjects and inclusion/exclusion criteria

Men or women aged 18–80 years with a history of mild to moderate essential hypertension (treated or untreated) were screened. Subjects with treated hypertension were required to withdraw from oral antihypertensive medications for 8–14 days prior to clevidipine administration, and to be off all antihypertensive medication for 7 days prior to study drug administration. To be eligible for study inclusion, subjects had to be assessed in the untreated state with systolic blood pressure (SBP) ≥140 mmHg and <200 mmHg and/or diastolic blood pressure (DBP) ≥95 mmHg and <115 mmHg, and heart rate (HR) <120 beats per minute (bpm), as described in the next section. Additional inclusion criteria included clinical laboratory test results within normal limits or with clinically insignificant abnormalities, negative illicit drug and alcohol screening tests, and if appropriate, negative urine pregnancy test, documented bilateral oophorectomy and/or hysterectomy, or postmenopausal status with cessation of menstruation for >2 years. Exclusion criteria included treatment with ≥5 oral antihypertensive agents, secondary hypertension, myocardial infarction or cerebrovascular event within 6 months, congestive heart failure or other severe cardiovascular disease, prior cerebral hemorrhage or intracranial tumor, liver failure or cirrhosis, intolerance to calcium channel blockers, allergy to soybean oil or egg lecithin, or any other condition warranting exclusion from the study per clinical judgment. Subjects unwilling to refrain from smoking, caffeine and alcohol and those involved in another study within 30 days were also excluded.

### Assessment for study eligibility based on BP and HR inclusion criteria

For subjects with untreated hypertension, BP and HR were obtained two or more times and at least 30 min apart on three separate days during the screening/run-in period. Subjects had to rest supine for at least 5 min before assessment. For study eligibility, on each occasion the average of the two BP measurements had to meet the inclusion criteria for SBP and/or DBP defined in the previous section, and HR had to be <120 bpm at every assessment. If the average SBP and/or DBP was above the upper limit of the inclusion criteria, the subject was withdrawn from the study and an appropriate antihypertensive regimen was established under the guidance of the primary care physician or physician investigator.

Subjects with treated hypertension discontinued their oral antihypertensive medications during the run-in period in the reverse order in which they had been prescribed, or, if the order was unknown, according to clinical judgment. During this period, BP and HR were monitored at clinic visits every 2–3 days, with measurements obtained after a minimum of 5 min supine rest. If SBP or DBP was above the upper limit of the infusion criteria at any clinic visit during screening, the same action was taken as described for untreated subjects. During the 7-day period when subjects were off all antihypertensive medication prior to study drug administration, subjects visited the clinic at least every 2–3 days. At each visit, BP and HR were obtained two or more times and at least 30 min apart after a minimum of 5 min supine rest per assessment. To be eligible for the study, each subject had to have three or more consecutive average SBP and DBP measurements on separate days that met the inclusion criteria defined in the previous section, including one average from assessments carried out on Day −1. Additionally, HR had to be <120 bpm at every assessment.

### Randomization and blinding

Eligible study subjects were allocated to one of four dosing cohorts and randomized to clevidipine or placebo within each cohort. It was planned that within each dosing cohort, 10 subjects would receive clevidipine and 3 would receive placebo. Subjects were replaced if necessary to ensure that at least 10 clevidipine-treated and 3 placebo-treated subjects per dosing cohort completed the study. Study drug infusions (clevidipine or placebo) were identical in appearance; to maintain the single blind, labels were covered while infusions were administered.

### Study treatment

Subjects were treated with IV clevidipine (0.5 mg/mL in 20 % lipid emulsion) or with placebo (Intralipid^®^ 20 % IV fat emulsion) in one of four dosing cohorts defined by clevidipine target doses of 2.0 mg/h, 4.0 mg/h, 8.0 mg/h, or 16.0 mg/h. Clevidipine was administered at an initial infusion rate of 2.0 mg/h to subjects in each cohort and force-titrated in doubling increments every 3 min to the target dose, as appropriate. Placebo was administered at the same rate as the corresponding dose of clevidipine. Clevidipine or placebo infusions were continued for 72 h once the final infusion rate had been achieved. Blood pressure changes were monitored using an automated sphygmomanometer.

Concomitant medications permitted during the run-in, treatment, and follow-up periods included non-prescription pain medications (without stimulants) used as per the package instructions and oral contraceptives; and during treatment and follow-up only, oral lorazepam 10 mg not exceeding once every 24 h at bedtime. Non-study drug medications and treatment procedures affecting BP were prohibited for 7 days prior to study drug administration, the treatment period, and 4 h following termination of study drug infusion. If alternative antihypertensive medication was required, the subject was withdrawn from the study.

All subjects could withdraw from the study at any time. All subjects could be withdrawn by the study physician at any time. Any subject withdrawn because of tachycardia or hypotension was to have study drug infusion down-titrated in halving increments prior to discontinuation. Any subject withdrawn due to hypertension was to have study drug infusion maintained during transition to oral antihypertensive(s). If withdrawn for safety concerns, subjects had vital signs monitored until vital signs were stable for at least 30 min.

### Study endpoints

The primary endpoint of the study was mean percentage change in SBP from baseline over the 72-h treatment period. Secondary study endpoints were mean percentage change in SBP from baseline over the first 4 h post-study drug infusion; clevidipine blood concentration over the 72-h treatment period through 60 min post-study drug infusion; the relationship between the time-matched, placebo-adjusted mean percentage change in SBP from baseline, and the mean blood concentration of clevidipine over the 72-h treatment period through 60 min post-study drug infusion; and the safety of prolonged (72 h) clevidipine infusion assessed according to clinical laboratory parameters, HR, electrocardiography (ECG), adverse events (AEs), and clinical evaluation.

### Pharmacodynamic assessments

During the treatment period, BP and HR were obtained pre-dose, then every 3 min during the titration phase to coincide with dose changes. After the target dose was reached, BP and HR were obtained at 5, 10, and 15 min and at 0.5, 2, 4, 8, 12, 16, 24, 30, 36, 42, 48, 54, 60, 66, and 72 h. After study drug termination, BP and HR were obtained at 2, 4, 6, 8, 12, 20, 30, and 60 min; then every 15 min for the next 3 h; then at 6 and 8 h. Subjects could resume their oral BP medication(s) 4 h after study drug termination in the order previously prescribed, or had an appropriate antihypertensive regimen established under physician guidance. On Day 7, BP and HR were measured to ensure that BP had returned to its pre-study baseline and had stabilized. If it had not stabilized, the subject was referred to his or her primary physician or had medications adjusted by the physician investigator. On all occasions, BP and HR were obtained after the subject had rested supine for at least 5 min. The BP cuff was not placed on the arm receiving study drug infusion. When BP and HR were scheduled to be obtained at the same time as a blood sample, the blood sample was drawn at the scheduled time point and BP and HR were assessed as soon as possible afterwards.

The dose–response relationship between percentage change from baseline in SBP and clevidipine infusion rate (mg/h) was also assessed, using the percentage change in SBP from baseline to the first SBP measurement after an increase in infusion rate (but before the next dose rate change) as the response value to the actual infusion rate.

Dose response was summarized using a random coefficient regression model, including intercept, slope, and adjustment for nonlinearity (square of infusion rate). The variance–covariance matrix of random effects was assumed to be unstructured. To eliminate the issue of co-linearity between slope and nonlinearity, the clevidipine infusion rate was standardized by subtracting the overall mean infusion rate prior to fitting the random coefficient regression model.

### Pharmacokinetic assessments

Blood samples for determining clevidipine blood concentrations were collected pre-dose and 0.5, 2, 4, 8, 12, 16, 24, 30, 36, 42, 48, 54, 60, 66, and 72 h following achievement of the target dose. Blood samples were also collected at 2, 4, 6, 8, 12, 20, 30, and 60 min after infusion cessation.

Samples were analyzed for concentration of clevidipine by liquid chromatography/mass spectrometry/mass spectrometry (LC/MS/MS) assay (Frontage Laboratories, Inc, Malvern, PA, USA); the lower limit of quantification was set at 0.2 ng/mL with an accuracy of 112 % and precision ( %CV) of 7.8 %. The calibration curves were linear in the range 0.20 to 500 ng/mL. Blood samples were not analyzed for placebo-treated subjects. Blood concentration–time data for clevidipine were analyzed by noncompartmental analysis using the software program WinNonlin^®^ Professional version 5.0.1 (Pharsight^®^ Corporation, Mountain View, CA, USA). Actual sampling times and actual total dose (mg) were used in the calculation of noncompartmental PK parameters.

C_max_ was obtained by visual inspection of the concentration–time profiles and is the highest observed value that was obtained. When feasible, C_ss_ was calculated by averaging the last four blood concentrations of clevidipine during the end phase of the infusion (average of the 54-, 60-, 66- and 72-h time points). AUC_0-t_ was estimated by a combination of linear trapezoidal methods on increasing concentrations and logarithmic trapezoidal methods on decreasing concentrations. The 73-h concentration value was set as “t” for this AUC_0–t_ calculation.

When feasible, λ_z_ was determined using unweighted linear regression analysis on at least three log-transformed concentrations visually assessed to be on the linear portion of the terminal slope, but not including the peak concentration. In general, objective selection of points included in the estimation of half-life required selection of those points that maximized R^2^ for the linear regression. The last measurable concentration was always included, unless the terminal data point appeared to be part of a new phase, or there was reason to believe that the last concentration was in error. When feasible, t_½_ was calculated as the ratio of log_e_2 to λ_z_ (t_½_ = 0.693/λ_z_).

### Safety assessments

Non-serious AEs were assessed and recorded from study drug initiation up to 8 h following study drug termination. Serious AEs were assessed from screening/run-in to Day 7 of the follow-up period. Blood samples were collected (after the subjects had fasted for at least 8 h) at screening, Day 4 and Day 7 for blood chemistry, hematology, and lipid panel tests. A 12-lead ECG was performed at each clinic visit during the screening/run-in period, then daily until discharge from the clinical research unit and at the Day 7 follow-up visit. A medical history was obtained during the screening/run-in period, and a physical examination was performed at the screening/run-in period and upon study check-in (Day −1).

### Statistical analysis

The planned sample size of 52 subjects was judged to be adequate for this PK/PD study without formal power calculation. The safety population was defined by protocol as all subjects who were dosed with any study drug. The per-protocol (PP) population was defined as all subjects who received 72 h of continuous treatment at the target dose and had blood samples for PK analysis obtained with correct methodology, and was designated as the primary population for BP, PK, and PD analyses, to allow analysis of data unaffected by incorrect blood sampling. Additionally, for better assessment of randomness, the intent-to-treat (ITT) population was defined as all randomized subjects.

In addition to analysis of pre-specified study endpoints, post-hoc analyses were performed. In order to more completely evaluate the potential for rebound hypertension over a longer period, mean percentage change in SBP and in DBP from baseline and placebo-corrected changes with respect to baseline were evaluated at 6 and 8 h following the cessation of study drug infusion. Descriptive statistics and graphs were used to summarize study data according to clevidipine dose level (2.0, 4.0, 8.0, 16.0 mg/h), and placebo; data from all placebo-treated subjects allocated to the four dosing cohorts were pooled.

The ECG parameters included ventricular HR, PR, QTS, and QT intervals. Laboratory parameters measured from different local laboratories, including blood hematology, serum chemistry, and lipid panels, were converted to conventional units and normalized to a standard set of reference ranges [11–13]. Change from baseline for each of the ECG and laboratory parameters was summarized descriptively at each time point.

## Results

### Study populations

Sixty-one hypertensive subjects were enrolled in the study, and received either clevidipine (*n* = 48) or placebo (*n* = 13), and comprise the ITT and Safety populations. Sixty subjects completed the study: one subject withdrew consent. The Per Protocol population (*n* = 53) consists of 40 clevidipine-treated subjects (7 subjects whose blood samples were drawn incorrectly and one subject who withdrew are excluded) and 13 placebo-treated subjects.

### Subject characteristics

Screening demographics and other baseline characteristics are summarized in Table 1. Subject medical history included co-morbidities common to this population.

**Table 1:** Screening demographics and other baseline characteristics (ITT population)

Parameter	Placebo	Clevidipine	Clevidipine	Clevidipine	Clevidipine	*P* value
		2.0 mg/h	4.0 mg/h	8.0 mg/h	16.0 mg/h	
	*n* = 13	*n* = 10	*n* = 10	*n* = 18	*n* = 10	
Mean age (SD), years	55 (12.6)	56 (15.3)	47 (11.2)	52 (12.8)	56 (10.4)	0.3866
Mean weight (SD), kg	88.1 (18.95)	87.4 (25.11)	105.3 (17.51)	93.6 (18.18)	99.9 (19.33)	0.1814
Mean BMI (SD), kg/m^2^	32.1 (5.64)	30.2 (4.43)	34.3 (5.85)	30.6 (5.52)	34.4 (7.05)	0.2542
Gender						
Male	7 (53.8 %)	5 (50.0 %)	7 (70.0 %)	14 (77.8 %)	7 (70.0 %)	0.5205
Female	6 (46.2 %)	5 (50.0 %)	3 (30.0 %)	4 (22.2 %)	3 (30.0 %)	
Ethnicity						
Hispanic	3 (23.1 %)	3 (30.0 %)	1 (10.0 %)	1 (5.6 %)	1 (10.0 %)	0.3881
Non-Hispanic	10 (76.9 %)	7 (70.0 %)	9 (90.0 %)	17 (94.4 %)	9 (90.0 %)	
Race						
White	9 (69.2 %)	7 (70.0 %)	7 (70.0 %)	12 (66.7 %)	8 (80.0 %)	0.9660
Black or African American	4 (30.8 %)	3 (30.0 %)	3 (30.0 %)	6 (33.3 %)	2 (20.0 %)	
Mean baseline SBP (SD), mmHg	151 (16.7)	152 (14.1)	143 (12.0)	150 (12.9)	155 (17.1)	0.4725
Mean baseline DBP (SD), mmHg	84 (4.1)	79 (10.1)	87 (15.8)	86 (12.9)	89 (7.7)	0.3760
Mean baseline HR (SD), bpm	66 (11.0)	64 (12.3)	82 (8.1)	67 (10.8)	73 (13.6)	0.0033
Hypertension	13 (100.0 %)	10 (100.0 %)	10 (100.0 %)	18 (100.0 %)	10 (100.0 %)	1.0
Number of subjects on prior antihypertensive medications ( %)	6 (46.2 %)	6 (60.0 %)	5 (50.0 %)	6 (33.3 %)	6 (60.0 %)	0.6029
Number of antihypertensive medications per subject, mean (median)	1.3 (1.0)	1.2 (1.0)	1.0 (1.0)	2.2 (2.0)	1.5 (1.5)	
Hospitalization for hypertension	1 (7.7 %)	0 (0.0 %)	1 (10.0 %)	0 (0.0 %)	1 (10.0 %)	0.4331
Diabetes (non-insulin-dependent)	2 (15.4 %)	3 (30.0 %)	3 (30.0 %)	3 (16.7 %)	3 (30.0 %)	0.7657
Transient ischemic attack	0 (0.0 %)	1 (10.0 %)	0 (0.0 %)	0 (0.0 %)	0 (0.0 %)	0.4918
Cigarette smoker	3 (23.1 %)	2 (20.0 %)	2 (20.0 %)	2 (11.1 %)	0 (0.0 %)	0.6889
COPD	0 (0.0 %)	0 (0.0 %)	0 (0.0 %)	0 (0.0 %)	1 (10.0 %)	0.4918
Dyslipidemia	2 (15.4 %)	5 (50.0 %)	1 (10.0 %)	3 (16.7 %)	2 (20.0 %)	0.2713

Table data expressed as number (percentage) unless otherwise noted

BMI = body mass index; SBP = systolic blood pressure; DBP = diastolic blood pressure; HR = heart rate; SD = standard deviation; COPD = chronic obstructive pulmonary disease

*P* values for screening demographics and baseline BP and heart rate are based on one-way ANOVA (continuous variables) or Chi-squared test (categorical data). *P* values for medical history are based on Fisher's exact test

No meaningful differences in oral antihypertensive regimens between cohorts were observed prior to the study start and following termination of study drug infusion.

### Pharmacodynamic evaluation

**Fig. 1:**
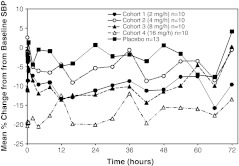
Mean percentage change from baseline in systolic blood pressure versus time over the full study drug infusion period for all clevidipine dosing cohorts (per-protocol population). *SBP* systolic blood pressure

**Fig. 2:**
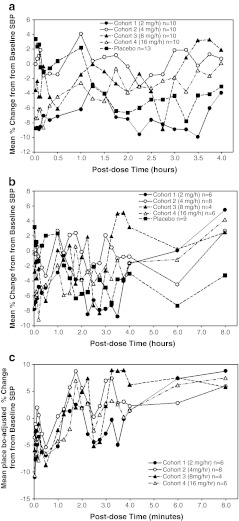
Mean percentage change from baseline in systolic blood pressure versus time after the end of study drug infusion (per-protocol population). **a** 0–4 h post-infusion. **b** 0–8 h post-infusion, excluding subjects who took oral antihypertensive medications within 8 h. **c** 0–8 h post-infusion (excluding subjects who took oral antihypertensive medications within 8 h) and adjusted for placebo response

All clevidipine dosing cohorts showed a larger percentage decrease in SBP from baseline compared with placebo (Fig. 1). The mean percentage change in SBP from baseline for the 4.0 mg/h, 8.0 mg/h, and 16.0 mg/h cohorts was dose-proportional. The 2.0 mg/h cohort showed a larger percentage decrease in SBP from baseline than the 4.0 mg/h cohort. A rapid onset of drug effect was observed for all clevidipine cohorts, with no evidence of a diminishing drug effect throughout the treatment period. From the end of infusion to 4 h post-infusion there was a rapid return to baseline SBP with no evidence of rebound (Fig. 2a). A rapid onset of DBP reduction, rapid return to baseline DBP, and no rebound in DBP were also shown in all dose cohorts.

A post-hoc analysis of mean percentage change from baseline in SBP for up to 8 h after the end of infusion was performed, with integration of additional BP safety monitoring assessments at 6 and 8 h. To avoid confounding the analysis, subjects were excluded if they restarted their oral antihypertensive therapy within 4 to 8 h after the end of infusion. There were nonsignificant trends toward higher than baseline blood pressures at 8 h (but not at 6 h) in the clevidipine-treated subjects, without an apparent dose–response relationship (Fig. 2b): oscillations in mean percentage change in SBP from baseline were noted from 9 % below to 6 % above baseline for 8 h after infusion end for the four clevidipine dose groups. Blood pressures in the placebo group were prominently below baseline at 6 and 8 h with mean percentage change in SBP remaining negative (i.e. below baseline). As a result of this negative trend, placebo-adjusted mean percentage change in SBP for the clevidipine dose groups shows a positive trend 6 and 8 h after cessation of clevidipine infusion (Fig. 2c). A similar exploratory analysis (not shown) showed no evidence of rebound in DBP for the full 8 h after the end of infusion.

### Pharmacokinetic evaluation

**Fig. 3:**
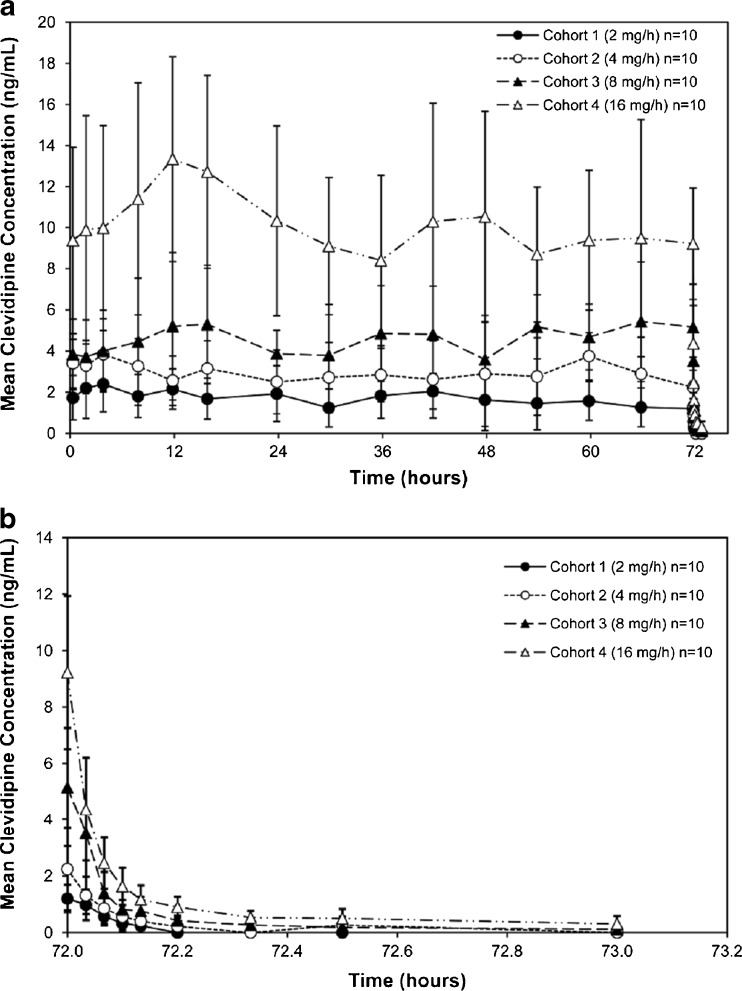
Mean clevidipine blood concentrations versus time

Mean clevidipine blood concentrations versus time were variable throughout the infusion period and decreased biexponentially after infusion cessation across all clevidipine dosing cohorts (Fig. 3). Following IV infusion, there was a less than dose-proportional increase in mean C_max_, C_ss_, and AUC_0-t_ between the clevidipine 2.0 mg/h cohort and the 16.0 mg/h cohort (Table 2). For an 8-fold increase in dose, there was an increase of approximately 4.7-fold in C_max_, ~6.7-fold in C_ss_, and ~5.9-fold in AUC_0-t_. Consequently, a decrease in dose-normalized C_max_ and a decrease in dose-normalized AUC_0-t_ were observed, as dose increased. Mean t_½_ ranged from 3 to 4 min in the initial decline phase and from 32 to 37 min in the later terminal phase, across all dose groups (Table 2). The calculated mean clearance for clevidipine was approximately 30 L/min, and was similar across all dose groups evaluated (Table 2).

**Table 2:** Pharmacokinetic parameters for clevidipine

Clevidipine infusion dosing cohort (mg/h)	*n*	C_max_ (ng/mL)	AUC_0-t_ (ng*h/mL)	C_ss_ (ng/mL)	CL (L/min)	t_½_ (earlier phase; min)	t_½_ (later phase; min)
2	10	3.36 (1.35)	122 (56)	1.37 (0.70)	33.2 (21.0)	4.18 (2.59)^a^	NC
4	10	5.17 (1.67)	211 (92)	3.00 (1.25)	26.1 (10.9)	3.28 (1.06)^a^	37.0 (29.9)^b^
8	10	7.68 (2.37)	327 (109)	5.12 (1.62)	28.5 (9.91)	3.16 (1.40)	32.4 (33.6)^a^
16	10	15.8 (4.01)	724 (246)	9.20 (3.37)	33.4 (14.7)	3.34 (0.96)	37.3 (21.7)^c^

Data expressed as mean (SD)

NC = Not calculated; AUC_0-t_ = area under the concentration–time curve from time zero to the last quantifiable concentration; C_max_ = maximum concentration; C_ss_ = steady-state concentration; CL = clearance. t_½_ = half-life

^a^ *n* = 8

^b^ *n* = 3

^c^ *n* = 9

### Pharmacodynamic and pharmacokinetic relationship

**Fig. 4:**
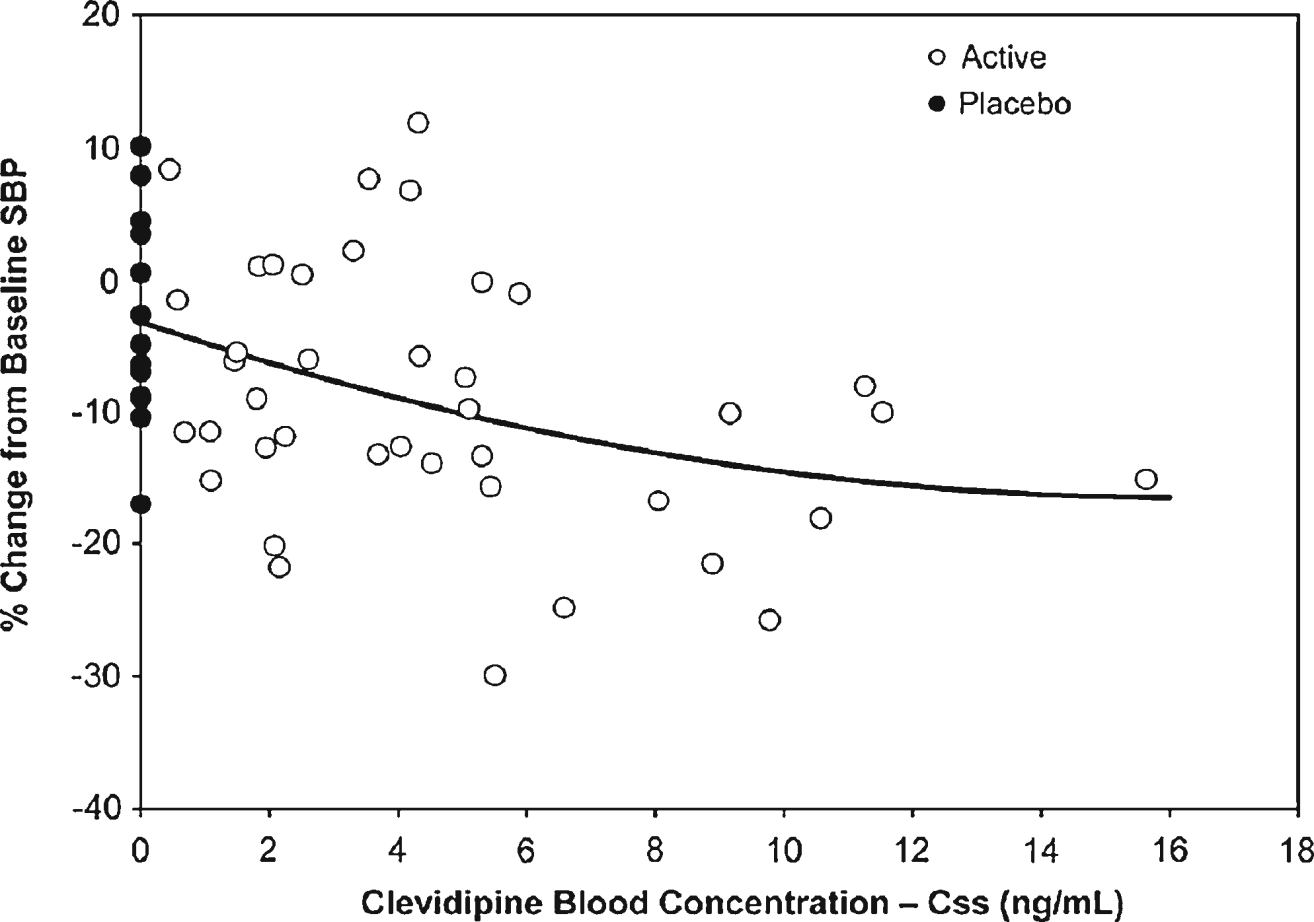
Clevidipine C_ss_ versus mean percentage change from baseline in systolic blood pressure at steady state, pooled across all cohorts

The individual C_ss_ values across cohorts were evaluated versus the respective matching percentage change from baseline SBP values. Although variability was observed, there was a visual trend toward a greater percentage decrease in SBP from baseline with increased steady-state blood concentrations of clevidipine (Fig. 4). The concentration–response curve was shallow and there was no apparent maximal response in change from baseline SBP over the range of C_ss_ values evaluated.

**Fig. 5:**
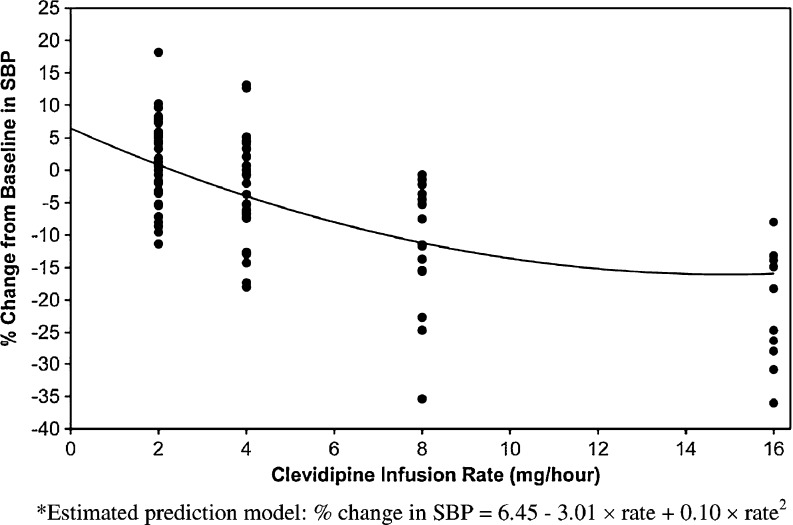
Model* prediction of systolic blood pressure dose–response (per protocol population)

A random coefficient regression model (Fig. 5) was used to estimate the dose–response relationship between percentage change from baseline in SBP and clevidipine infusion rate (mg/h). The maximum percentage change from baseline in SBP predicted by the model (Table 3) was associated with a clevidipine infusion rate of 16 mg/h and was estimated at −22 % (lower limit of 95 % confidence interval [CI] −22.32 %, −9.53 %; point estimate: −15.93 %). An estimated clevidipine infusion rate of approximately 10 mg/h was needed to achieve half this rate. Based on the ITT population, the maximum percentage change from baseline was estimated at −25 % (lower limit of 95 % CI of −24.15 %, −9.39 %; point estimate: −16.77 %). Table 3 also predicts that every 1 mg/h increase in the clevidipine infusion rate was associated with a 1 % to 1.5 % decrease in SBP. That is, given a mean baseline SBP of 150 mmHg, an increase of approximately 1–2 mg/h in the infusion rate of clevidipine may produce an additional 2–4 mmHg decrease in SBP from baseline.

**Table 3:** Prediction of dose response (per protocol population)

Infusion rate	Prediction of % change in SBP (95 % CI)
2 mg/h	0.83 (−1.16, 2.82)
4 mg/h	−3.99 (−6.29, −1.69)
8 mg/h	−11.20 (−14.61, −7.80)
10 mg/h	−13.59 (−17.23, −9.95)
16 mg/h	−15.93 (−22.32, −9.53)

### Tolerance

To evaluate the potential for subjects to develop acute tolerance to prolonged infusions of clevidipine, plots of individual clevidipine blood concentration versus percentage change from baseline SBP during the 72-h drug infusion, and up to 1 h post-drug infusion were generated for the clevidipine 16.0 mg/h cohort. Based on visual inference, there was no evidence of hysteresis (different percentage change in SBP from baseline at the same clevidipine concentration during infusion or washout) and thus no evidence of acute tolerance during the observation period. The change from baseline SBP remained relatively constant during the infusion period, and a trend toward increasing change from baseline SBP with increased clevidipine concentration was observed, providing further evidence that tolerance does not occur over a 72-h clevidipine infusion.

### Safety

All subjects received clevidipine or placebo as a 72-h IV infusion with the exception of one subject who withdrew from the study. Forty-eight subjects comprised the safety population and received total clevidipine doses between 144 mg and 1,152.7 mg. Safety assessments based on clinical laboratory test results were similar among all dose groups. There were no treatment- or dose-related trends in the blood chemistry or hematology results during the study. One subject receiving clevidipine 8.0 mg/h had an alanine aminotransferase (ALT) level of 55 U/L (standard normal range 13 to 40 U/L) on Day 7. Nine clevidipine-treated subjects had one or more post-baseline triglyceride levels greater than 500 mg/dL (of potential clinical significance); 2 of these subjects had elevated triglycerides at baseline. Of the remaining 7 subjects, 1 (8.0 mg/h cohort) had triglyceride levels >500 mg/dL on Day 4, and 6 subjects had elevated triglycerides on Days 7/8, but not at 0 to 8 h post-cessation of clevidipine infusion. No changes or findings associated with study drug safety were noted from the 12-lead ECGs for this study.

Forty-three out of 61 subjects experienced one or more AEs, generating a total of 112 reported AEs (Table 4). The majority were assessed as mild or moderate and 1 (back pain in a placebo-treated subject) was assessed as severe. The number of AEs and subjects reporting AEs were similar across treatment groups. The most commonly reported treatment-emergent AEs were headache, infusion site reaction, and infusion site swelling (Table 5).

**Table 4:** Adverse events by treatment group (safety population)

Adverse Events	Clevidipine *N* = 48				Placebo	Total
	2 mg/h	4 mg/h	8 mg/h	16 mg/h		
	*n* = 10	*n* = 10	*n* = 18	*n* = 10	*n* = 13	*n* = 61
Number of adverse events	20	15	23	26	28	112
Number of subjects with AEs ( %)	9 (90)	7 (70)	12 (67)	8 (80)	7 (54)	43 (70)
Severity, number of subjects ( %)						
Mild	8 (80)	5 (50)	12 (67)	8 (80)	7 (54)	40 (66)
Moderate	3 (30)	5 (50)	1 (6)	1 (10)	2 (15)	12 (20)
Severe	0	0	0	0	1 (8)	1 (2)

**Table 5:** Most common treatment-emergent adverse events (TEAEs; safety population)

Most common TEAEs	Clevidipine *N* = 48	Placebo *N* = 13
	*n* ( %)	*n* ( %)
Headache	18 (37.5)	2 (15.4)
Infusion site reaction	6 (12.5)	4 (30.8)
Infusion site swelling	5 (10.4)	1 (7.7)

No deaths or serious AEs occurred during this study. One subject treated with clevidipine (8.0 mg/h) withdrew from the study after experiencing three AEs (generalized facial pressure, headache, and nausea) that were assessed as mild and treatment-related. The AEs were reported and resolved on Day 1, and the subject withdrew from the study on Day 2.

## Discussion

The results of this study support the use of IV clevidipine in BP management for up to 72 h. In subjects with mild to moderate essential hypertension, 72-h continuous infusion did not affect the PK or PD characteristics of clevidipine over the dose range of 2.0 to 16.0 mg/h. No evidence of tolerance to the drug effect over a 72-h infusion, or of rebound hypertension for 4 or 6 h after termination of infusion was observed. Furthermore, no hysteresis was seen after examining each individual plot of clevidipine concentrations versus change from baseline in SBP for the high-dose cohort (16.0 mg/h), indicating that acute tolerance did not occur during a 72-h infusion. Clevidipine demonstrated a favorable safety and tolerability profile when administered as a prolonged continuous IV infusion.

Previous studies of clevidipine infusions over shorter durations have demonstrated a linear relationship between blood concentrations at steady-state and dose rate [14, 15]. Based on post-hoc analysis with a random coefficient regression model, the maximal effect of clevidipine in blood pressure reduction was estimated at 25 % of baseline SBP at 16 mg/h, and the estimated infusion rate needed to achieve half this maximal effect was estimated to be approximately 10 mg/h.

The elimination half-lives of clevidipine observed after 72 h are similar to those obtained after a 20-min infusion in healthy volunteers at a final dose rate of 3.2 μg/min/kg (approximately 16 mg/h), reported as 0.6 and 2.3 min for the initial two rapid elimination phases (accounting for about 95 % of the area under the concentration–time curve) and 16 min for the terminal phase [8–10]. In the present study, because no blood samples were obtained within the first 2 min after infusion cessation, the t_½_ values assessed for the 16 mg/h dosing cohort probably represent the second and terminal decline phases of the clevidipine blood concentration time profile. The clearance of clevidipine was very high and similar across all dose groups evaluated. The clearance is much greater than cardiac output and is a consequence of the rapid and complete metabolism that occurs within a short time of administration.

Clevidipine is metabolized through hydrolysis of the ester linkage by esterases in the blood and extravascular tissues to inactive metabolites, accounting for its ultra-short elimination half-life [16, 17]. Rapid clearance from the blood and a lack of accumulation in tissues suggest that clevidipine is unlikely to exert long-term effects after the infusion is stopped, such as the minimal elevations in serum potassium and ALT observed 4 days after discontinuation of infusion in the present study.

The lack of a known mechanism also makes it less plausible that a true rebound effect would be observed 8 h after stopping infusion, given that no evidence of rebound hypertension is observed 6 h after infusion cessation and no clear dose–response relationship at 8 h can be determined. In this study, the placebo group showed a negative mean percentage change in SBP after stopping the infusion. No specific reason for this observation is known, although untreated SBP may be expected to fluctuate in both directions, and a placebo effect resulting in sustained SBP reduction in subjects with acute hypertension has been observed in previous studies [6, 7]. The observed mean reduction was primarily driven by two placebo patients having large SBP reductions, and may simply have been due to reduced anxiety as they completed their last study SBP assessments. A post-hoc comparison of SBPs for subjects receiving prior antihypertensive medication with those not having prior medication confirmed that there were no meaningful differences in these subpopulations during study drug infusion and following termination of study drug infusion. The larger percentage decrease in SBP from baseline with the 2.0 mg/h cohort than with the 4.0 mg/h cohort may possibly be related to the lower baseline SBP in the 4.0 mg/h cohort (143 mmHg vs 152 mmHg for the 2.0 mg/h cohort).

Of the 9 subjects who had one or more post-baseline normalized triglycerides that were greater than 500 mg/dL, 6 subjects had potentially clinically significant elevations on Days 7 and 8. These elevations were not thought to be caused by clevidipine administration as the infusion had ended 3 or 4 days prior to the collection of blood samples for clinical chemistry evaluation. Two of the remaining subjects had elevated triglycerides at baseline, one of whom was shown to have dyslipidemia prior to entering the study. The one subject with elevated triglyceride levels on Day 4 was receiving clevidipine infusion at the same time that the blood sample was drawn. Since clevidipine emulsion and Intralipid^®^ contain 20 % soybean oil (composed primarily of triglycerides), this finding is understandable. Overall, the changes in triglycerides were sporadic, not dose dependent, and, in the majority of subjects, not thought to be drug related. In previous clinical studies with clevidipine emulsion, there were no reports of adverse clinical effects due to the Intralipid^®^ formulation. However, patients with disorders of lipid metabolism may be less able to rapidly clear fat from circulation, thereby resulting in transient triglyceride elevations.

After 72-h continuous infusion with IV clevidipine 2.0 to 16.0 mg/h in subjects with mild to moderate essential hypertension, no tolerance to drug effects over a 72-h continuous infusion, and no rebound hypertension for up to 6 h after infusion cessation was observed over all clevidipine dose levels.
